# Low Body Mass Index and Blood Loss in Primary Total Hip Arthroplasty: Results from 236 Consecutive Ankylosing Spondylitis Patients

**DOI:** 10.1155/2014/742393

**Published:** 2014-05-14

**Authors:** Jinzhu Zhao, Jia Li, Wei Zheng, Denghui Liu, Xiaofeng Sun, Weidong Xu

**Affiliations:** ^1^Department of Orthopedics, Changhai Hospital, Second Military Medical University, 168 Changhai Road, Shanghai 200433, China; ^2^Department of Orthopedics, No. 401 Hospital, Jinan Military Region of PLA, Qingdao, Shandong 266071, China

## Abstract

*Objective*. To evaluate the effect of low body mass index (BMI) on blood loss during primary total hip arthroplasty (THA) in ankylosing spondylitis (AS) patients. *Methods*. Two hundred seventy-seven consecutive AS patients who underwent primary THA were retrospectively studied. The patients were divided by BMI into an underweight group (BMI < 18.5 kg/m^2^) and a normal weight group (18.5 kg/m^2^ < BMI < 25 kg/m^2^). Demographics, perioperative laboratory values, intraoperative data, blood loss, transfusion rate, transfusion reactions, surgical complications, hospitalization cost, and length of stay (LOS) were collected and analyzed. *Results*. Of 277 AS patients, 236 were eligible for inclusion in the study. A total of 91 (39%) patients were underweight. The hidden blood loss, transfusion rate, transfusion reactions, and hospitalization cost in the underweight group were significantly higher than those in the normal weight group. *Conclusions*. For AS patients, BMI appears to be correlated with blood loss during primary THA. Compared with patients of normal weight, low BMI patients have the potential to suffer more postoperative hidden blood loss and to require a higher transfusion rate.

## 1. Introduction

Hip involvement is common in ankylosing spondylitis (AS) patients and can often be disabling due to ankylosis [1]. In cases of end-stage hip involvement, total hip arthroplasty (THA) is an effective treatment [2–6].

THA is a procedure that frequently results in significant blood loss. Previous literature has reported that 19%–69% of patients who underwent THA had to have blood transfusions [7–9].

Many risk factors for blood loss in THA have been reported, and body mass index (BMI), which has drawn the attention of researchers worldwide, seems to be a particularly important risk factor. Obese patients experience more blood loss during THA than patients of normal weight [10–14]. However, some researchers have reported that there is not a significant correlation between BMI and blood loss during THA [15–17]. After reviewing the literature extensively, we found that most previous studies focused on the correlation between obesity and blood loss during THA. Few studies on the correlation between low BMI and blood loss during THA are available.

According to our clinical observations, low BMI was more common in AS hip involvement patients than in those who received THA for other causes, such as osteoarthritis, developmental dysplasia of the hip (DDH), and femoral neck fracture. We hypothesized that low BMI may influence blood loss during THA. Therefore, a retrospective study was conducted on AS patients to examine the correlation between low BMI and blood loss during primary THA.

## 2. Materials and Methods

### 2.1. Patients

A set of 277 AS patients who underwent primary THA from December, 2006, to June, 2012, were included in the present study. Forty-one patients were excluded due to incomplete data (*n* = 10) or because they were undergoing bilateral procedures simultaneous to THA (*n* = 31). Finally, 236 patients were eligible for study inclusion.

### 2.2. Surgical Indications, Technique, and Perioperative Management

The surgical indications were as follows: Bath Ankylosing Spondylitis Radiology Hip Index (BASRI-hip) ≥3, obvious impairment of hip function, and no surgical contraindications. All patients were evaluated by both surgeons and anesthesiologists one month before surgery. Nonsteroidal anti-inflammatory drugs (NSAIDs) were interrupted two weeks before surgery. BASRI-hip was scored by two blinded experienced readers according to preoperative anteroposterior pelvic radiographs. Dual energy X-ray absorptiometry (DXA) was employed to measure bone mineral density (BMD) of the hip if there were any suspicious manifestations of low BMD in pelvic radiographs. Osteoporosis was defined based on WHO criteria [18]. According to the DXA results, the implants were selected (cement implants for osteoporotic patients and cementless implants for nonosteoporotic patients).

The operations were performed by the same surgery team; the surgical technique was standardized, and general anesthesia was used for all patients. All procedures were carried out with the patients in the lateral position. The posterolateral approach and the same hemostasis techniques were used for all patients. To reduce postoperative blood loss, external wound compression was used without drainage for 48 hours after surgery.

To prevent infection, cefuroxime sodium was routinely applied for 24 hours perioperatively. The postoperative venous thromboembolic prophylaxis included mechanical prophylaxis with thromboembolic disease stockings and was commenced immediately after surgery and continued for 5 weeks.

### 2.3. Transfusion Management and Calculation of Blood Loss

The transfusion triggers were a hemoglobin concentration less than 80 g/L and a hematocrit below 25%. For patients > 60 years old, a hemoglobin concentration below 100 g/L was a transfusion trigger. Allogeneic blood transfusion was performed when it was required based on the triggers, and salvage of autologous blood was not used preoperatively.

The BV was calculated according to the formula described by Nadler et al. [19]. Perioperative total blood loss was estimated based on the Hb balance method [20]. Intraoperative blood loss was determined by the assistant as the sum of gauze weighted plus the difference between the suction and irrigation volumes. The postoperative hidden blood loss was calculated as the difference between the total blood loss and the intraoperative blood loss. The ratios of total blood loss and BV, intraoperative blood loss and BV, and postoperative hidden blood loss and BV were calculated. Finally, we used the ratio of blood loss and BV as the criterion for evaluating blood loss for individuals in this study.

### 2.4. Assessment

The following data were collected from all patients: age, gender, height, weight, disease duration, Bath Ankylosing Spondylitis Disease Activity Index (BASDAI), Bath Ankylosing Spondylitis Functional Index (BASFI), BASRI-hip score, osteoporosis status, preoperative thromboplastin time (TT), preoperative activated partial thromboplastin time (APTT), preoperative platelet (PLT) level, preoperative albumin (ALB) level, perioperative hemoglobin (Hb, preoperative and fifth day postoperative) levels, perioperative hematocrit (HCT, preoperative and fifth day postoperative) levels, length of external wound compression time, length of stay (LOS), and hospitalization cost. Moreover, additional operative data were collected: acetabular cup diameter, operating time, intraoperative blood loss, allogeneic blood transfusion rate and volume, transfusion reactions and operative complications, such as early infection, poor healing of the incision, and dislocation.

BMI was calculated based on the World Health Organization (WHO) criteria, and the BMI of the 236 patients in this study ranged from 14.2 to 24.9 kg/m^2^. The patients were divided into two groups based on weight. The underweight group included 91 patients (BMI < 18.5 kg/m^2^), and the normal weight group included 145 patients (18.5 kg/m^2^ < BMI < 25 kg/m^2^).

### 2.5. Statistical Analysis

The statistical analyses were performed using SPSS 17.0 software. Student's *t*-test was used for quantitative variables, and the chi-square test was used for qualitative variables. Differences were considered statistically significant with *P* < 0.05.

## 3. Results

Two hundred and thirty-six patients were identified, of which 91 (39%) were underweight. The demographics and anthropometry of the study patients are described in Table 1; there was a statistically significant difference in BV between the two groups. No statistically significant difference was found between the underweight group and the normal weight group in mean age and sex distribution.

The preoperative laboratory values, such as TT, APTT, PLT, Hb, HCT, and ALB, showed no statistically significant difference between the two groups. Regarding disease duration, there was no statistically significant difference in BASDAI and BASRI-hip scores between the two groups. The mean BASFI score was higher in the low BMI group. The analysis of the preoperative BMD showed that 15 osteoporotic patients were in the low BMI group, while 10 osteoporotic patients were in the normal weight group; this difference was statistically significant (Table 2).

Intraoperative data, blood loss, and transfusion rate are described in Table 3. The mean operating time and the diameter of the acetabular cup showed no statistically significant difference between the two groups. The ratio of total blood loss and BV in the low BMI group was significantly higher than in the normal group. The ratio of intraoperative blood loss and BV had no statistically significant difference between the two groups. However, the ratio of hidden blood loss and BV in the low BMI group was significantly higher than that in the normal weight group. The transfusion rate in the underweight group was higher than that in the normal weight group, and the mean transfusion volume (RBC transfused) did not show a statistically significant difference between the groups. The external wound compression time was shorter in the low BMI group than in the normal weight group.

The main transfusion reaction symptoms in the present study were tetter and fever, and there were more transfusion reactions in the underweight group than in the normal weight group. There was no statistically significant difference between the two groups in regard to surgical complications (Table 4).

**Figure 1:**
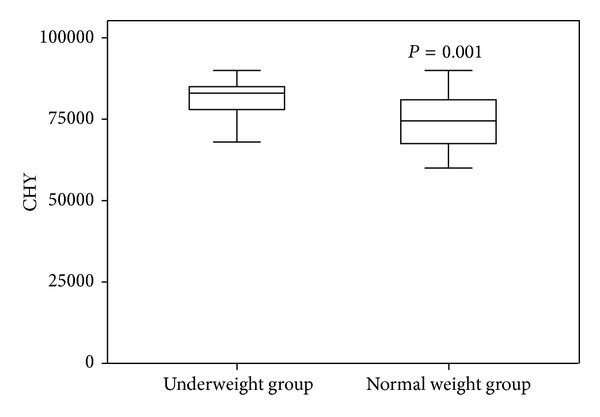
Comparison of in-hospital costs between underweight group and normal group.

**Figure 2:**
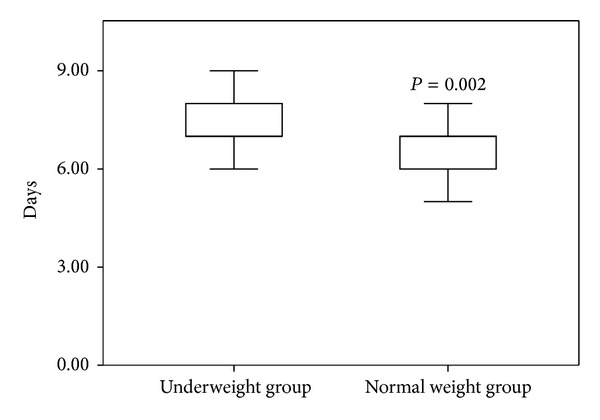
Comparison of the length of hospital stay between underweight group and normal group.

The mean overall hospitalization cost was 81087 (5688) CNY in the underweight group and 74575 (7805) CNY in the normal weight group; the difference between the groups was statistically significant (Figure 1). The LOS was significantly different between the two groups (Figure 2).

## 4. Discussion

This retrospective study analyzed whether low BMI influences blood loss during THA in AS patients. Our results show that there are statistically significant differences between the underweight group and the normal weight group in postoperative hidden blood loss and transfusion rate during THA. In addition, transfusion reactions, LOS, and hospitalization cost were higher in the underweight group than in the normal weight group.

To our knowledge, few studies on the epidemiology of BMI in AS patients are available. In the present study, 39% of patients were underweight. Some previous studies mostly examined obese patients [21–23]. The difference in the BMI distribution between our study and others may be the result of the following factors. First, all patients in our study had end-stage hip involvement; in those patients osteoporosis is frequent and disease duration is longer. Second, AS is a chronic disease that requires long-term treatment, and the socioeconomic status of AS patients could affect treatment efficacy and influence the general condition of AS patients. China is a developing country; therefore, the difference in the developmental level of the Chinese economy and the economies of western countries may explain the higher frequency of underweight cases in our study than in other studies mentioned above.

The ratio of blood loss and BV is more objective than the amount of blood loss when estimating the influences of blood loss on the body; this is particularly true in underweight and obese patients. In the present study, we used the ratio of blood loss and BV as the criterion for evaluating blood loss. Our results suggested that the intraoperative blood loss was similar in the two groups, but the hidden blood loss and total blood loss were higher in the underweight group than in the normal weight group. The preoperative baseline characteristics, blood coagulation function (TT, APTT, and PLT), and nutritional status (Hb and ALB) of the two groups were similar in our study. Therefore, there must be other factors that caused the difference in postoperative hidden blood loss between the two groups.

Wound compression has been used after THA in some studies [24] and was found to significantly reduce the need for transfusion. In the present study, a bellyband was used for external wound compression in all cases to reduce postoperative blood loss and transfusion. The shorter external wound compression time may have been a factor responsible for the higher hidden blood loss and transfusion rate in the low BMI group. Further prospective studies should be performed to estimate the relationship between external wound compression time and blood loss in THA.

Several studies have shown that low BMI is associated with low BMD in AS [25–29]. These studies suggest a high prevalence of low BMD and a 13% to 16% prevalence of osteoporosis in AS patients within 10 years after diagnosis. Our results confirmed that osteoporosis was more frequent in the low BMI group than in the normal weight group. The higher prevalence of osteoporosis may contribute to low BMI and to more postoperative hidden blood loss. As we know, the initial stability of the implant depends on the press-fit fixation in THA, and osteoporotic patients are susceptible to fracture. More postoperative hidden blood loss could occur as a result of more periprosthetic microfractures in the low BMI group than in the normal weight group. This assumption requires further study for confirmation.

The transfusion rate and transfusion reactions were higher in the underweight group. The increased transfusion rate in the underweight cases was caused by higher total blood loss and higher postoperative hidden blood loss in the low BMI group, but the mechanism of the increased transfusion reactions in the low BMI group is not entirely clear. This may indicate the low compensative ability and poor immune response to stresses, such as surgical strike, blood loss, and allogeneic blood transfusion, in underweight patients. The higher overall cost and longer LOS in the underweight group may be caused by the higher postoperative hidden blood loss, higher transfusion rate, and the increased transfusion reactions in the low BMI group.

Blood management is an important perioperative issue in arthroplasty. To reduce blood loss and transfusion rate perioperatively, techniques, such as controlled hypotension, intraoperative blood salvage, autologous blood transfusion, wound compression, and administration of antifibrinolytic agents, have been used [30, 31]. Among these measures, the use of tranexamic acid (TXA) may be the most blood sparing method after THA [32]. TXA is a synthetic antifibrinolytic drug that is a competitive inhibitor of plasminogen and can decrease fibrinolysis. Numerous studies have reported the effectiveness of TXA in THA [32–35]; they found that TXA can reduce both blood loss and transfusion rates. At the same time, the incidence of thromboembolic complications after THA did not increase in those patients who were given TXA.

The limitations of the present study are as follows. First, this is a retrospective study. Second, the sample size was limited, and we did not include an obese patient group. More research will be performed in the future to determine the risk factors for low BMI in end-stage AS, the effect of low BMD on blood loss in THA, and so forth.

## 5. Conclusions

We demonstrated that low BMI influences blood loss during THA in AS patients. Compared with the normal weight patients, the underweight patients suffered from more postoperative hidden blood loss, and the transfusion rate of the low BMI group was higher. In addition, transfusion reactions, LOS, and hospitalization cost were higher in the low BMI group than in the normal weight group.

## Figures and Tables

**Table 1:** Demographics and anthropometry of the study patients.

	Underweight group	Normal weight group	*P *
Number of patients	91	145	
Age (y)*	36 (8)	37 (8)	0.485
Male/female	76/15	126/19	0.472^†^
BMI (kg/m^2^)*	17.0 (1.3)	21.9 (1.6)	<0.001
BV (L)*	3.92 (0.65)	4.56 (0.56)	<0.001

*Mean (SD). ^†^Chi-square test.

BMI: body mass index; BV: blood volume.

**Table 2:** Preoperative baseline characteristics of 236 AS patients.

	Underweight group	Normal weight group	*P*
Disease duration (y)*	13 (4)	12 (6)	0.100
BASDAI*	5.2 (0.8)	5.3 (0.8)	0.334
BASFI	5.5 (0.7)	5.0 (0.8)	<0.001
BASRI-hip 4/3	56/35	87/58	0.841^†^
Osteoporosis/nonosteoporosis	15/91	10/145	0.020^†^
TT (S)*	13.1 (0.6)	13.1 (0.8)	0.876
APTT (S)*	36.8 (2.9)	36.9 (3.3)	0.808
Hb-pre (g/L)*	127 (16)	128 (15)	0.227
HCT-pre (%)*	38 (4)	39 (4)	0.090
PLT (×10^9^/L)*	252 (62)	248 (59)	0.527
ALB (g/L)*	37 (4)	37 (3)	0.073

*Mean (SD). ^†^Chi-square test.

BASDAI: Bath Ankylosing Spondylitis Disease Activity Index; BASFI: Bath Ankylosing Spondylitis Functional Index; BASRI-hip: Bath Ankylosing Spondylitis Radiology Hip Index; TT: thromboplastin time; APTT: activated partial thromboplastin time; Hb-pre: preoperative hemoglobin; HCT: hematocrit; PLT: platelet; ALB: albumin.

**Table 3:** Intraoperative data, blood loss, and transfusion.

	Underweight group	Normal weight group	*P*
Operating time (min)*	60 (11)	61 (8)	0.984
Diameter of acetabular cup (mm)*	52.0 (2.2)	52.4 (2.0)	0.092
Intrablood loss/BV (%)*	10 (1)	10 (2)	0.277
Hidden blood loss/BV (%)*	15 (1)	13 (3)	<0.001
CTBL/BV (%)*	25 (2)	23 (4)	<0.001
Transfusion rate	75/91	98/145	0.012^†^
RBCs transfused (U)*	1.9 (0.2)	2.0 (0.1)	0.235
Length of external wound compression time (h)	35 (7)	43 (6)	<0.001

*Mean (SD). ^†^Chi-square test. CTBL: calculated total blood loss; BV: blood volume; RBC: red blood cell.

**Table 4:** Complications during hospitalization.

	Underweight group	Normal weight group	*P*
Transfusion reactions	17/75	7/98	0.003^†^
Surgical complications			
Poor healing of incision	6/91	8/145	0.733^†^
Early infection	0	0	
Dislocation	0	0	

^†^Chi-square test.
